# A rare case of concurrent left ventricular aneurysm and ventricular septal rupture complicating an inferior myocardial infarction: a case report

**DOI:** 10.11604/pamj.2023.45.74.39612

**Published:** 2023-06-02

**Authors:** Mariem Drissa, Fares Azaiez, Fekher Jaoued, Rouaida Khelifa, Elyes Lagha, Rim Ben Romdhane, Rami Tlili, Youssef Ben Ameur

**Affiliations:** 1 Department of Cardiology, Mongi Slim Hospital, Tunis, Tunisia

**Keywords:** Myocardial infarction, ventricular septal rupture, left ventricular aneurysm, case report

## Abstract

Complications following acute myocardial infarction (MI) such as ventricular septal rupture (VSR) and left ventricular (LV) aneurysm are rare and can be dreadful. Their simultaneous presence in the same patient is extremely rare. We aimed to present a rare case of concomitant association of ventricular aneurysm and VSR complicating an inferior myocardial infarction. We report the unusual case of Mr. A. D, a 63-year-old, active smoker, with a history of diabetes mellitus and hypertension, admitted for the management of inferior MI within 6 days. The MI was complicated by an LV aneurysm in the inferoposterior and the inferoseptal walls associated with a VSR in the inferoseptal wall. The patient had only signs of right heart failure on admission. This observation illustrates on the one hand the rarity of the association of VSR and LV aneurysm after an inferior myocardial infarction, and on the other hand the possibility of founding them at an early stage of MI without any signs of cardiogenic shock.

## Introduction

Complications following acute myocardial infarction (MI) such as ventricular septal rupture (VSR) are rare and dreadful, usually occurring within a week of the inaugural episode and its incidence is accounting for less than 1% of total cases in the era of myocardial revascularization [1]. In addition, the left ventricular (LV) aneurysm post-MI has an incidence of 3.5% to 38% and is usually associated with anterior MI. The occurrence of these complications is generally associated with high mortality. The simultaneous presence of a VSR and an LV aneurysm in the same patient is extremely rare.

In the current case report, we aimed to present a rare case of a concomitant association of ventricular aneurysm and VSR complicating inferior MI.

## Patient and observation

**Patient’s information:** Mr AD, a 63-year-old patient, an active smoker with a consumption estimated at 30 packyears, with a history of diabetes mellitus on oral antidiabetics, a history of hypertension, was admitted to the cardiology departments of the Mongi Slim of Marsa Hospital for the management of an inferior ST-segment elevation myocardial infarction (STEMI) complicated by right heart failure. The patient reported the notion of intense continuous inaugural epigastralgia, 6 days before admission, neglected by the patient and treated by gastric bandages and analgesics taken on his own.

**Clinical findings:** on admission, the patient was conscious, with tachycardia with a heart rate at 120 bpm, and blood pressure at 120/70 mmHg. On cardiac auscultation, the patient had a wheel-spoke holosystolic murmur at the left lower sternal border. The patient presented signs and symptoms of right ventricular failure including painful hepatomegaly, hepatojugular reflux, and turgor of the jugular veins. Apart from tachycardia, he had no signs of left heart failure. The patient did not show peripheral signs of shock.

**Figure 1:**
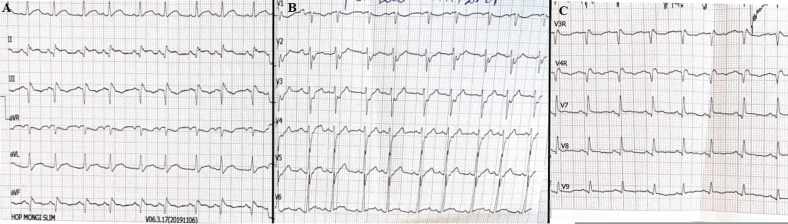
(A,B,C) electrocardiogram (ECG) of the patient showing sinus rhythm narrow QRS, ST segment elevation in the inferior leads associated with necrotic Q wave and negative T wave

**Diagnostic assessment:** the electrocardiogram (ECG) showed a sinus rhythm with a normal axis, narrow QRS, ST-segment elevation in the inferior leads associated with necrotic Q wave and negative T wave, and ST segment depression in the apicolateral leads (Figure 1). The laboratory tests of the patient showed acute renal failure (blood creatinine = 380 μmol/l), hepatic cytolysis, and a spontaneously low prothrombin time (PT) at 51%. The rest of the biological assessment is unremarkable.

**Figure 2:**
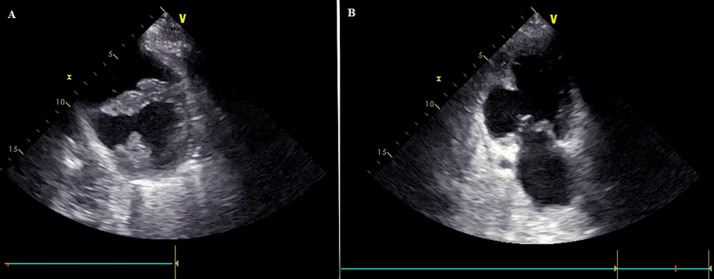
echocardiography findings: A) parasternal short-axis view showing the aneurysm; B) apical 2-chamber view showing the aneurysm

**Figure 3:**
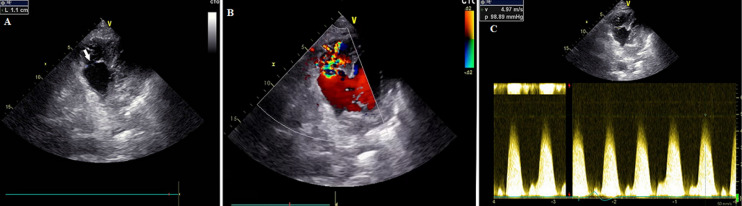
A) parasternal short-axis view showing the ventricular septal rupture; B) parasternal short-axis view with color-Doppler showing the flow across ventricular septal rupture (VSR); C) continuous Doppler showing restrictive flow

A transthoracic echography was performed, showing a moderately altered left ventricular ejection fraction (LVEF) at 45%, with akinesia of the apical segment of the inferoseptal wall. It objectivated also a thin-walled aneurysmal formation with a globular structure, in the median and basal segments of the inferoposterior and the inferoseptal walls and having a systolic dyskinetic contraction. This aneurysm with globular structure (36 mm × 38 mm) communicates with the left ventricle through a wide neck. It was connected to the right ventricle (RV) through a perforation measured at 11 mm and located in the full wall of the aneurysm, framed with a ventricular septal perforation. A left-to-right shunt was noted with a maximum speed of 4.97 m/s. Therefore, the RV is of normal size with moderate systolic dysfunction and the systolic pulmonary artery pressure was estimated at 60 mmHg. A schematic view of the aneurysm and VSR is shown in Figure 2 and Figure 3.

**Figure 4:**
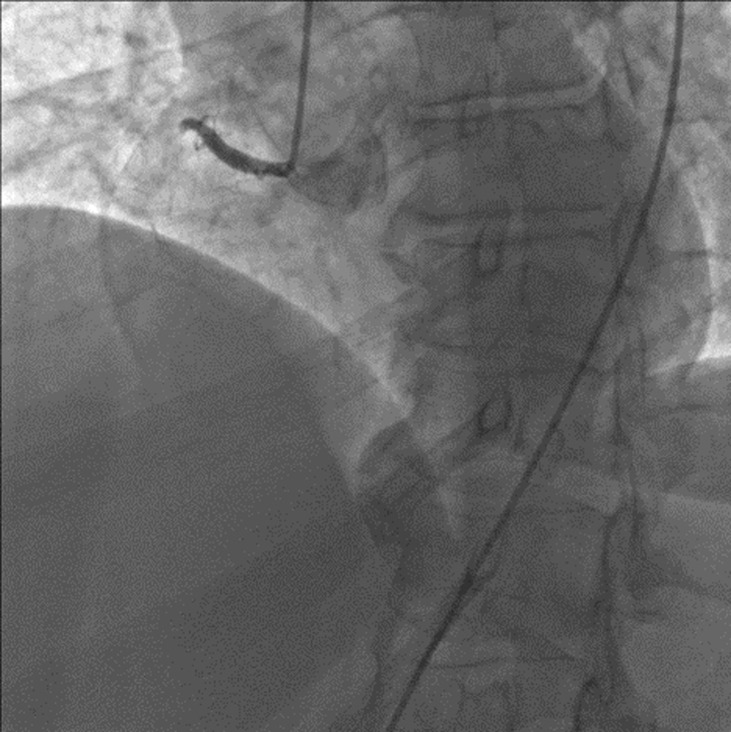
the patient's coronary angiography shows a total acute occlusion of the right coronary artery at its proximal part

Coronary angiography was conducted on the patient. It revealed a total acute occlusion of the proximal segment of the right coronary artery (RCA) (Figure 4). Furthermore, non-significant lesions were found in the left coronary arteries.

**Therapeutic intervention:** we recommended an emergency coronary artery bypass graft (CABG) surgery of the RCA along with aneurysmectomy and ventricular septal repair. The patient was subsequently referred to the cardiovascular surgery department for further treatment.

**Follow-up and outcomes:** the evolution was marked by the deterioration of the hemodynamic state of the patient and the appearance of cardiogenic shock. Unfortunately, the patient died before being operated on.

**Patient’s perspective:** we had the opportunity to discuss the treatment and management of the patient, Mr A. D, with him and his family. Mr. A. D expressed his gratitude for the care and attention he received during his hospitalization. He acknowledged the complexity of his case and understood the limitations faced by the medical team in successfully treating his condition.

**Informed consent:** this was obtained from Mr. A. D prior to any procedures or interventions. The purpose, risks, and potential benefits were explained, and Mr. A. D willingly consented to the recommended treatments.

## Discussion

The LV aneurysm represents a frequent complication of STEMI [2] and its incidence has declined due to emergent reperfusion strategies for acute MI. The type of LV aneurysm is usually a true aneurysm which is defined as an abnormal protrusion of the ventricular wall and it can be formed in 5 days in an infarct-related artery [3]. Aneurysms occur approximately four times more often at the apex and anterior wall than the inferoposterior wall [2] which is in accord with our case that shows an inferoposterior wall aneurysm which is quite rare. VSR is another rare but fatal complication following MI. Although the incidence of this dreaded complication has come down in this reperfusion era, it still has high mortality and should not be overlooked particularly in patients presenting late.

Female gender, chronic kidney disease, and advanced age are the main risk factors for the occurrence of VSR [4]. It generally occurs earlier during the post-infarction period. The location of VSR depends on the type of STEMI and occurs approximately three times more often at the anterior or apical portion of the interventricular septum (IVS) than the inferoposterior IVS [4]. Our case is unique since the VSR location was less common than the typical sitting. Therefore, the concurrence of VSR and LV aneurysm, especially inferior LV aneurysm, in the same patient is rare. The hemodynamic status of these patients depends on many factors such as the RV infarction and the size of the defect. It varies from hemodynamic stability to a hemodynamic collapse but the VSR is more prevalent in patients with cardiogenic shocks [5]. The mechanical complications of STEMI often have fulminant and catastrophic manifestations in patients, and the existence of two concurrent MI-induced mechanical complications and a relatively non-fulminant course is rare.

This finding was observed in our case who had initially both complications and developed within 6 days symptoms of right heart failure without showing any signs of cardiogenic shock. Some reports in the literature have demonstrated similar cases of VSR and LV aneurysms [6,7].

Recently, several cases have reported the occurrence of concurrent inferoposterior LV aneurysms and VSR secondary to an inferior MI [6,8,9]. But our case describes a unique inferior MI complication in many ways. First, the occurrence of true LV aneurysm in the inferoposterior and the inferoseptal walls is uncommon. Second, VSR rarely complicates inferior MI and its location in the inferoseptal wall is rare. Third, the concurrence of true LV aneurysm and VSR is reported only in several cases. Fourth, our patient presented a rare case of two MI complications without presenting symptoms of cardiogenic shock on admission. Finally, the occurrence of these complications secondary to an inferior MI within 6 days in the post-infarction period has not been reported unlike the cases quoted above.

## Conclusion

Our case reported an unusual and rare case of concurrent true inferoposterior LV aneurysm and VSR after inferior MI observed at an early stage of acute coronary syndrome. The concurrence of these complications is rare, especially in the post-infarction period, and worsens the prognosis.
